# Apoproteins E, A-I, and SAA in Macrophage Pathobiology Related to Atherogenesis

**DOI:** 10.3389/fphar.2019.00536

**Published:** 2019-05-21

**Authors:** Godfrey S. Getz, Catherine A. Reardon

**Affiliations:** ^1^Department of Pathology, The University of Chicago, Chicago, IL, United States; ^2^Ben May Department for Cancer Research, The University of Chicago, Chicago, IL, United States

**Keywords:** macrophage, apoE, apoA-I, SAA, cholesterol efflux, oxidation, mimetic peptides, atherosclerosis

## Abstract

Macrophages are core cellular elements of both early and advanced atherosclerosis. They take up modified lipoproteins and become lipid-loaded foam cells and secrete factors that influence other cell types in the artery wall involved in atherogenesis. Apoproteins E, AI, and SAA are all found on HDL which can enter the artery wall. In addition, apoE is synthesized by macrophages. These three apoproteins can promote cholesterol efflux from lipid-loaded macrophages and have other functions that modulate macrophage biology. Mimetic peptides based on the sequence or structure of these apoproteins replicate some of these properties and are potential therapeutic agents for the treatment of atherosclerosis to reduce cardiovascular diseases.

## Introduction

Macrophages are a core cellular element of both early and advanced atherosclerotic lesions in humans and experimental animal models. Many of the macrophages of lesions are derived from blood monocytes entering regions of the arterial wall lined with activated endothelial cells. Most studies of the involvement of macrophages in atherosclerosis are concerned with several features of macrophage pathobiology: the ingress of monocytes into lesions and their differentiation into macrophages; the conversion of macrophages to lipid loaded foam cells; with the genes the macrophages express in the context of the lesions that influence other cells in the lesion including endothelial cells, smooth muscle cells and cells of the adaptive immune system; and with the egress of macrophages from the lesion. The accumulation of macrophages in arterial lesions is not only due to the balance of the ingress and egress of monocytes/macrophages, but also consequent on local macrophage proliferation (Robbins et al., 2013; Williams et al., 2018). In early atherosclerotic lesions the macrophage foam cell containing stored cholesteryl ester droplets is the most obvious biomarker of the process. These features have directed attention to the lipid metabolism of these cells.

The detailed examination of the evolution of lesions is not possible in humans as the mechanistic study of the atherogenic process is difficult to study directly and individual subject exhibit quite different genotypes. Instead the evolution of human atherosclerosis is largely inferred from the analysis of atherosclerotic lesions obtained from patients, living or dead. One of the best examples of this approach is represented by the PDAY study, an autopsy-based study of young individuals deceased as a result of accidents (McMahan et al., 2008). In this study, the extent and distribution of lesions in the vasculature in the subjects was correlated with risk factors (e.g., gender, smoking, hyperlipidemia, hypertension, and diabetes). Given the limitations of the refined examination of human lesion development, attention has turned to animal models of atherosclerosis, with most studies in the last decades utilizing murine models lacking either the apoE gene (*Apoe*) or the LDL receptor (*Ldlr*) gene (Getz and Reardon, 2012). Indeed, since first being described in 1992 (Plump et al., 1992; Zhang et al., 1992) the *Apoe^-/-^* mouse has become the favored model for the study of experimental murine atherosclerosis (Getz and Reardon, 2016). ApoE is a multifunctional protein (Getz and Reardon, 2009) that is normally present on circulating lipoproteins, where it functions as a ligand for lipoprotein uptake, particularly for the removal of intestinal derived chylomicron remnants and hepatic derived VLDL remnants. As a result, *Apoe^-/-^* mice are hyperlipidemia even while being fed a low-fat chow diet. This hyperlipidemia is further increased by feeding a high fat, high cholesterol diet Western type diet.

## The Influence of Apoe on Macrophages and Monocytes

### Macrophage Expression of ApoE Reduces Atherosclerosis

ApoE is expressed in many cell types, particularly hepatocytes and macrophages. The only cells that do not express significant levels of this apoprotein are enterocytes (Driscoll and Getz, 1984). The significance of the apoE produced by macrophages for atherogenesis has been highlighted by bone marrow transplantation experiments. Macrophages are a major apoE-producing cell derived from bone marrow precursors, and implicit in these transplantation studies is that the macrophages are the operative cells responsible for the reported results. When *Apoe^-/-^* mice were transplanted with wild type bone marrow, apoE levels in the plasma increased and the hyperlipidemia and atherosclerosis exhibited a notable reduction, despite only low levels of apoE in the plasma (Linton et al., 1995). Unfortunately, the co-ordinate reduction of both does not allow for the assessment of the contribution of the macrophage derived apoE to the attenuation of atherosclerosis independent of effects on plasma lipids. However, there are a number of experiments in *Apoe^-/-^* mice that point to an independent influence of macrophage derived apoE on atherosclerosis. It appears that the plasma concentration of apoE required for rescue of the dyslipidemia is higher than that associated with the reduction in lesion formation. In one study, retrovirus mediated apoE expression in macrophages rescued early atherosclerotic lesion development with little effect on plasma lipids (Hasty et al., 1999). In these experiments the plasma level of apoE was only about 1% of levels in wild type mice. In another study (Bellosta et al., 1995) the visna virus LTR was employed to drive the transgenic expression of human apoE in macrophages in *Apoe^-/-^* mice. Mice with a range of plasma lipids and apoE levels were obtained. When a subgroup of animals was selected that had essentially the same blood lipid levels and lipoprotein profile as *Apoe^-/-^* control mice, a significant reduction in lesions was noted in the transgenic mice, again implying that the expression of apoE by macrophages was capable of attenuating lesion development independent of effects on blood lipids. Compatible with this interpretation is the increased early lesion formation observed when bone marrow from *Apoe^-/-^* mice was transplanted into high fat, high cholesterol fed wild type mice (Fazio et al., 1997).

### Macrophage ApoE and Cholesterol Efflux

**FIGURE 1:**
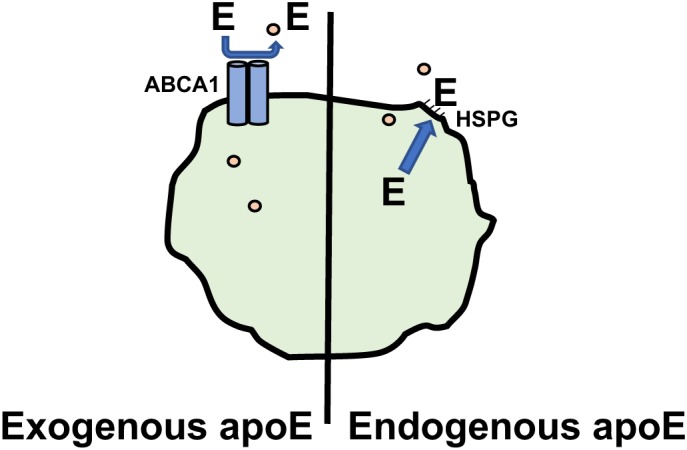
Promotion of Cholesterol efflux by apoE. Macrophage cholesterol (∘) efflux promoted by exogenous apoE is dependent on ABCA1. Cholesterol efflux promoted by endogenous apoE involves apoE associating with the plasma membrane via heparin sulfate proteoglycans(HSPG) and is ABCA1 independent.

The function of apoE in macrophages that is thought to be most important for its anti-atherogenic role is its ability to enhance cholesterol efflux from the arterial macrophages, thereby reducing their lipid burden and the subsequent downstream production of macrophage products, such as pro-inflammatory cytokines and chemokines that promote atherogenesis (Table 1). We have recently reviewed the role of apoE in cellular cholesterol efflux and reverse cholesterol transport (Getz and Reardon, 2018). Most studies of cholesterol efflux *in vitro* and *in vivo* use mouse macrophage cell lines, such as J774A.1 or RAW264.7 cells. However, the role of apoE in promoting cholesterol efflux may be under appreciated since, unlike tissue macrophages including those found in atherosclerotic lesions, these cell lines do not express apoE. Advantage has been taken of the absence of apoE expression by J774 cells to examine differences between exogenous and endogenous apoE in promoting cholesterol efflux. For this, J774 cells were transfected to express apoE under the control of a non-cholesterol responsive promoter. Comparing these cells with untransfected cells it was shown that endogenous apoE is more effective in promoting cholesterol efflux than is exogenous apoE added to the media (Lin et al., 1999). It appears that the efflux promoted by endogenous apoE is qualitatively different than that seen with exogenous apoE (Figure 1). Unlike exogenous apoE, endogenous apoE-mediated efflux is not ABCA1 dependent (Huang et al., 2001). Instead, the apoE produced by the cells is found associated with the plasma membrane, bound to either the LDL receptor, heparan sulfate proteoglycan or membrane lipids (Zhao and Mazzone, 1999; Lin et al., 2001). In humans there are three isoforms of apoE, designated apoE2, apoE3, and apoE4. ApoE2 and apoE4 differ from the most prevalent apoE3 isoform by single amino acid; residues 112 and 158 are cysteine and arginine, respectively in apoE3, both residues are cysteines in apoE2 and both are arginines in apoE4. ApoE2 has significantly lower binding affinity for the LDL receptor, while apoE4 has slightly higher binding affinity. Human apoE isoform replacement mice have been created in which each human apoE isoform replaces the endogenous murine apoE. When LDL receptor expression in macrophages from the gene replacement mice was upregulated by simvastatin treatment, apoE protein secretion and cholesterol efflux promoted by apoE4 was reduced while that of apoE2 was unaffected, reflecting their affinity for the LDL receptor (Lucic et al., 2007).

**Table 1:** Macrophage Related Functions of ApoE, ApoA-I, and SAA.

Apoprotein	Macrophage Related Function
ApoE	Synthesized by macrophages
	Promotes cholesterol efflux
	Regulates monocytosis
	Anti-inflammatory
	Antiatherogenic
ApoA-I	Promotes cholesterol efflux
	Myeloperoxidase mediated modifications reduces efflux capacity
	Antiatherogenic
SAA	Synthesized by macrophages
	Inhibits HDL mediated cholesterol efflux
	Proatherogenic

Reverse cholesterol transport is a process by which excess cholesterol in cells, including macrophages, is effluxed and transported via plasma lipoproteins to the liver for excretion in feces. This pathway is frequently examined *in vivo* using radiolabeled cholesterol loaded macrophages to follow the transport of the cholesterol from the macrophages to the feces. Consistent with the *in vitro* cellular cholesterol efflux results reported in the preceding paragraph, macrophages deficient in apoE are not as effective as wild type macrophages in supporting reverse cholesterol transport *in vivo* (Zanotti et al., 2011).

### Regulation of Macrophage ApoE Expression

As macrophage apoE plays a role in cholesterol efflux, it is of interest to understand the regulation of its expression in macrophages. ApoE production increases as monocytes differentiate into macrophages (Werb et al., 1986). In experiments with mouse peritoneal macrophages and human THP1 cells differentiated into macrophages, cholesterol loading of the cells in culture upregulates apoE mRNA and apoE protein secretion (Mazzone et al., 1987, 1989). On the other hand, macrophages derived from human monocytes do not exhibit an increase in apoE synthesis on cholesterol loading but endogenous levels are sufficient to enable cholesterol efflux (Zhang et al., 1996). A more recent study showed a similar up regulation of apoE mRNA in peritoneal macrophages harvested from *Ldlr^-/-^* mice fed a Western type diet containing 1.25% cholesterol (Spann et al., 2012). This appears to be attributable to activation of the liver X receptor (LXR) by the desmosterol that accumulates in the cholesterol loaded macrophages. The cholesterol mediated induction of apoE gene transcription, but not the basal level of apoE gene transcription, is abolished in the absence of LXR nuclear receptors (Laffitte et al., 2001). The results of Spann et al contrast with the proteomic analysis of proteins secreted from lipid-loaded peritoneal macrophage of *Ldlr^-/-^* mice fed the same Western type diet (Becker et al., 2010). Forty-six proteins were differentially expressed by peritoneal macrophages from chow and Western diet fed mice. Among these differentially expressed proteins, apoE secretion was profoundly decreased along with other proteins that have been shown to influence atherogenesis like MFGE8 (lactadherin), lipoprotein lipase, and LRP (LDL receptor related protein) from macrophages of Western type diet fed *Ldlr^-/-^* mice. ApoE appears to be a regulator of this protein network, as a different and attenuated pattern of proteins responsive to cholesterol loading was noted in macrophages from *Apoe^-/-^* mice. Since Western type diet fed mice are insulin resistant as well as hyperlipidemia, further studies examined the response of the macrophage protein network in the presence and absence of insulin resistance. ApoE and 8 other members of this macrophage protein network were dysregulated in the presence of insulin resistance via a mechanism dependent on IFNγ and independent of changes in their transcript levels. In addition, using *Ldlr-/-* mice fed high cholesterol diets with (insulin resistant) and without (insulin sensitive) high fat content, IFNγ was shown to be an important driver of atherosclerosis in mice with insulin resistance (Reardon et al., 2018). IFNγ modulation of the protein network was also observed in vessel wall macrophages. IFNγ is generally thought to be pro-atherogenic by promoting foam cell formation by stimulating the cell surface expression of scavenger receptors and by polarizing macrophages toward the pro-inflammatory M1 subset (Boshuizen and de Winther, 2015). The reduction in the expression of apoE and other proteins in this network may also contribute to the pro-atherogenic role of IFNγ, especially in the setting of insulin resistance. Parenthetically, it is noteworthy that peritoneal and bone marrow derived macrophages are not phenotypically identical, at least with respect to their response to oxidized LDL (Bisgaard et al., 2016). Clearly, despite much work on the role of apoE in macrophage biology, much remains to be clarified.

### ApoE and Monocytosis

It is now clear that monocytosis is a risk factor for the development of atherosclerotic cardiovascular disease as well as myocardial infarctions (Murphy and Tall, 2016; Swirski et al., 2016). This increased risk is related to dysfunction of cholesterol homeostasis, at least at the level of monocyte progenitor cells in the bone marrow and extramedullary sites such as the spleen. There are two major subsets of circulating monocytes that are distinguished by the level of Ly6C on their cell surface. The pro-inflammatory Ly6C^hi^ monocytes dominate in the monocytosis associated with hypercholesterolemia and these are the cells that preferentially infiltrate the artery wall and become lesional macrophages (Swirski et al., 2007). Tall and collaborators have explored the role of apoE in the expansion of monocyte precursor pools (Murphy et al., 2011). In a seminal prior study, they showed increased proliferation of hematopoietic stem and progenitor cells (HSPCs) when enriched in cellular cholesterol (Yvan-Charvet et al., 2010). Cholesterol accumulation was enhanced in this study because of an inability of the cells to efflux the sterol as a result of engineered deficiency of the ABC transporters ABCA1 and ABCG1. Subsequent studies demonstrated that the most efficient removal of cholesterol from the HSPCs requires the interaction of cell autonomous apoE (rather than circulating apoE) with the cell surface ABC transporters. This was shown in competitive bone marrow transplant experiments. The cell surface of the cholesterol loaded HSPCs are enriched in the common β-subunit of the IL-3/GM-CSF receptors. GM-CSF is produced by macrophages, T cells and by innate response activator B cells. The latter cells are expanded in secondary lymphoid organs in the context of hypercholesterolemia (Hilgendorf et al., 2014).

In much of the literature it is assumed that the macrophage is loaded with lipid once sequestered in the atherosclerotic lesion. However, recently it has been suggested that murine monocytes may acquire their initial lipid load while still in the circulation (Xu et al., 2015). Hypercholesterolemia in *Apoe^-/-^* mice results in lipid loading of blood monocytes, producing foamy monocytes. The foamy monocytes are positive for the expression of CD36 and CD11c, which correspond to Ly6C^lo^ monocytes. These monocytes were shown to enter nascent atherosclerotic lesions. This seems to be contrary to the prevailing literature, which concluded that the inflammatory Ly6C^hi^ monocytes represent the major monocyte subclass contributing to the evolving atherosclerotic plaque (Swirski et al., 2007). Of course, it is necessary to consider the stage of atherogenesis under study. Interestingly, Combadiere et al. (2008) reported that the extent of aortic root lesions in *Apoe^-/-^* mice correlated not only with total blood monocyte levels, but also with the level of Ly6C^lo^ monocytes. To what extent this is a function of the prosurvival signals exhibited by the CX3CR1 expressed by Ly6C^lo^ monocytes remains to be established (Landsman et al., 2009). In the normal artery wall, the resident macrophages mainly have an M2-like phenotype and are found in the adventitia. In early foam cell lesions in the *Apoe^-/-^* model, M2-like and M1-like macrophages are present in approximately equal numbers, while in advanced lesions M1-like macrophages predominate (Khallou-Laschet et al., 2010; Koltsova et al., 2013). The two subsets are not evenly distributed in human atherosclerotic lesions. M1-like macrophages are located in the shoulders and in the vicinity of the necrotic core that are associated with plaque rupture, with few M2 macrophages in these regions (Stoger et al., 2012). Khallou-Laschet et al. (2010) have suggested that M2 macrophages of the early lesions may be converted to M1 macrophages as the lesions progress, though this suggestion is not universally accepted (Peled and Fisher, 2014). The precise origin and role of the major individual monocyte/macrophage subsets in the evolution of the atherosclerotic plaque is not fully clarified despite a good deal of effort from several laboratories.

### Other Anti-atherogenic Functions of ApoE

While apoE has a major role in the regulation of macrophage cholesterol homeostasis, it also has other anti-atherogenic activities. ApoE is considered to be anti-inflammatory. One mechanism by which apoE may exert its anti-inflammatory function is by promoting the dominance of M2 macrophages, operating through the engagement of either the cell surface VLDL receptor or the apoE receptor 2 on macrophages (Baitsch et al., 2011). This is mediated in part by activating p38 MAP kinase signaling. Ly6C^lo^ monocytes express higher levels of apoE than do Ly6C^hi^ monocytes (Li et al., 2015). This probably extends also to their derived macrophages. M2 macrophages are also relatively enriched in the efferocytosis bridge molecule MerTK (DeBerge et al., 2017), suggesting that these cells in particular may be responsible for the uptake of apoptotic cells derived from free cholesterol overloaded pro-inflammatory macrophages. The pro-inflammatory M1 macrophages express high levels of NFκB while the M2 macrophages are rich in LXR and PPARγ that drive the production of apoE and the ABC transporters responsible for cholesterol efflux (Adamson and Leitinger, 2011). ApoE has also been shown to reduce lipid oxidation and the activation of endothelial cells, suppress innate immunity (Bouchareychas and Raffai, 2018) and reduce the migration and proliferation of smooth muscle cells in the intima (Swertfeger and Hui, 2001; Swertfeger et al., 2002). Recently an additional mechanism has been described accounting for the anti-inflammatory action of apoE (Li et al., 2015). In macrophages apoE induces miR-146a, which inhibits TRAF6 and IRAK 1 and hence NFκB activation. Thus, macrophage apoE has a multitude of actions that can impact atherogenesis only some of which are dependent on its influence on lipid homeostasis.

## Macrophage Myeloperoxidase Reduces the Cholesterol Efflux Promoting Ability of Apoa-I

ApoA-I is the major apoprotein found on HDL and one of the major anti-atherogenic functions of apoA-I/HDL is the promotion of cholesterol efflux and reverse cholesterol transport. They also have anti-inflammatory and anti-oxidative properties. Unlike apoE, no significant amount of apoA-I is produced by macrophages. However, plasma HDL can enter the artery wall to promote cholesterol efflux following interaction with ABCG1 on macrophages foam cells. In addition, small amounts of apoA-I may be displaced from the HDL and exist as lipid-poor particles that can promote cholesterol efflux from macrophage foam cells via interaction with ABCA1 to generate nascent HDL or pre-β HDL. These nascent HDL particles can promote further cholesterol efflux from macrophages following interaction with cell surface ABCG1 (Rosenson et al., 2016). Macrophages can influence the functionality of apoA-I. Macrophages, especially M2 macrophages, as well as neutrophils and monocytes release myeloperoxidase, an enzyme that has the capacity to oxidize methionine, tryptophan, and tyrosine residues in apoA-I. The myeloperoxidase oxidized apoA-I exhibits reduced cholesterol efflux capacity both in culture and *in vivo* (Zheng et al., 2004; Hewing et al., 2014), likely due to impaired interaction with ABCA1. Oxidation of tryptophan 72 appears to account for about 50% of its impaired function (Huang et al., 2014). When oxidized apoA-I is added to the plasma, the modified apoprotein does not bind well to HDL and is found in the lipid-poor fraction (Hewing et al., 2014). Lipid-poor apoA-I is more rapidly cleared from the plasma than is HDL associated apoA-I and this rapid removal could contribute to the low efficacy of modified apoA-I in promoting cholesterol efflux *in vivo* and to the relatively low extent of modified apoprotein found in the plasma. Interestingly, tryptophan 72 oxidized apoA-I is present in lipid-poor form in human atherosclerotic plaques at ∼1,000-fold higher levels than in the plasma. Tyrosine 166 in apoA-I is nitrated by myeloperoxidase and, like the oxidized tryptophan 72 containing protein, lipid-poor apoA-I containing this myeloperoxidase modified amino acid is enriched in the arterial wall lesions compared to the plasma (DiDonato et al., 2014). Thus, it is likely that, at least from the point of view of atherosclerosis, lipid-poor apoA-I is oxidized by macrophage derived myeloperoxidase in the artery wall resulting in impaired reverse cholesterol transport.

## The Influence of Saa on Macrophages

Serum amyloid A (SAA) and apoE have some broad similarities in properties, though not in detailed functions. They both contain multiple amphipathic helices, though different in overall structure. There are multiple isoforms of both apoE and SAA in humans, but in other species only SAA has multiple isoforms. Both proteins are lipoprotein associated; VLDL and HDL for apoE, and predominantly HDL for SAA. They are both primarily synthesized by the liver, though other cells, including macrophages, are capable of producing the proteins. Finally, both have the capacity to bind proteoglycans. While the range of concentrations of apoE in the plasma is modest, that of SAA is dramatic. It is present in plasma at quite low levels under basal conditions but is greatly increased in conditions of acute inflammation, the so-called acute phase reaction. In situations of chronic inflammation, such as atherosclerosis, plasma levels are modestly increased over basal levels. Indeed, its level in the plasma of patients with cardiovascular disease is useful as a biomarker of cardiovascular disease risk, at least as useful as C-reactive protein (Johnson et al., 2004).

The SAA isoforms are encoded by four genes. SAA1 and SAA2 are acute phase proteins. Their synthesis is stimulated by the cytokines IL-6 and TNFα and their plasma levels are notably elevated (∼1,000 fold) in acute inflammation. SAA1 and SAA2 are neighboring genes that are coordinately transcribed and the mature proteins differ in only 6 of 104 residues. In humans SAA4 is constitutively expressed and SAA3 is a pseudogene. However, in mice SAA3 is expressed, mostly in adipose tissue. Modeling of SAA1 based on structural studies describes a protein that has 4 helices that appear to have different functions. Helices 1 and 3 bind to HDL, helix 2 serves as a bridge between HDL and fibronectin and laminin and helix 4 serves as a bridge between HDL and proteoglycans (Frame and Gursky, 2017). While SAA1/2 are primarily associated with HDL in the acute phase, SAA turns over more rapidly than apoA-I and apoA-II, the major proteins of HDL, suggesting that the lipoprotein does not turnover as an intact particle (Kim et al., 2016).

### SAA and Reverse Cholesterol Transport

During acute inflammation there is a reduction in the reverse cholesterol transfer of macrophage cholesterol via the plasma to feces (McGillicuddy et al., 2009), but this reduction is accounted for by SAA in acute phase HDL only to a limited extent (de Beer et al., 2013). Scavenger receptor class B type I (SR-BI) is a cell surface receptor responsible for selective cholesteryl ester uptake from HDL and both HDL associated SAA and lipid-poor SAA bind to this receptor. But lipid-poor SAA inhibits the ability of SR-BI to promote selective cholesteryl ester uptake (Cai et al., 2005). In accord with the limited role of SAA in reverse cholesterol transport, knocking out SR-BI in mice has limited impact on reverse cholesterol transport (Wang et al., 2007). Thus, these pathways do not have a large quantitative role on the reduced reverse cholesterol transport observed in acute inflammatory states. Given the complexity of the proteome of HDL (Pamir et al., 2016), and the large changes in the composition of acute phase HDL (Vaisar et al., 2015), as well as in LCAT and in ABC transporters during the acute phase (Feingold and Grunfeld, 2010), other possibilities may be entertained. Nonetheless, the study of HDL from several inbred mouse strains under basal conditions revealed an inverse correlation of SAA1 levels on the HDL and its ability to promote ABCA1 dependent cholesterol efflux (Pamir et al., 2016).

### Pro-inflammatory Properties of SAA

Lipid-poor SAA has multiple pro-inflammatory actions that are mediated by a variety of cell surface receptors, including TLR2, TLR4, CD36, FPR2, RAGE, and P2XY (Eklund et al., 2012). The activation of macrophages by SAA stimulates the release of IL-8 and MCP-1 that function to attract neutrophils and monocytes, respectively into sites of inflammation. The secretion of other cytokines and factors known to promote atherogenesis are also increased. The lipid-poor SAA-mediated increase in IL-1β secretion from macrophages appears to be due to increased potassium efflux, cathepsin B activation and reactive oxygen species generation leading to the activation of the NLRP3 inflammasome (Shridas et al., 2018). All of these effects are attenuated by the addition of HDL, possibly due to the sequestering of the lipid-poor SAA. How lipid-poor SAA is generated in tissues such as the artery wall is not clear since little lipid-poor SAA is detected in plasma.

SAA plays a role in the retention of HDL in the arterial wall of atherosclerotic *Apoe^-/-^* and *Ldlr^-/-^* mice (O’Brien et al., 2005). SAA containing HDL is retained by binding to proteoglycan, such as biglycan. This results in segregation of the HDL from the cell surface of the macrophage foam cells of the lesion and hence a lower capacity to mediate cholesterol efflux from these cells. Consistent with this, HDL derived from SAA knockout mice has a much lower capacity for binding vessel wall proteoglycans and an enhanced cholesterol efflux potential (Chiba et al., 2011). Thus, the cholesterol efflux capacity of the intravascular HDL containing SAA is reduced. ApoE is also able to bind to proteoglycan, but in the context of the inflammation associated with atherosclerosis, HDL carries more SAA than apoE.

### SAA and Atherosclerosis

Based on the pro-inflammatory properties of SAA discussed above, one would expect that the overexpression of SAA would augment atherosclerosis and its removal would reduce lesion development. Indeed, lentivirus-mediated overexpression of murine SAA1 increased total aortic atherosclerosis and aortic root lesion in *Apoe^-/-^* mice (Dong et al., 2011). This was correlated with increased macrophage content of the lesions and an elevation of MCP-1 expression. The development of mice lacking the two major acute phase SAAs (*Saa1/2^-/-^* mice) has facilitated further studies on the role of SAA. Contrary to expectations, the absence of SAA1/2 in chow fed *Apoe^-/-^* mice had no effect on atherosclerosis when the vessels were examined at 50 weeks of age (De Beer et al., 2014). The absence of SAA1/2 in the *Ldlr^-/-^* model fed the Western type diet revealed a reduction of early lesion development in the ascending aortic arch (after 6 weeks of diet), which was no longer evident when the diet was extended to 12 weeks (Krishack et al., 2015). No changes were seen in the other arterial sites at either time. Reciprocal transplantation between *Ldlr^-/-^* and *Ldlr^-/-^ Saa1/2^-/-^* mice indicates that SAA derived from both systemic production and bone marrow derived cells participate in the early atherosclerosis phenotype. A reduction in atherosclerosis was also observed when the SAA receptor FPR2/ALX was knocked out in the *Ldlr^-/-^* background (Petri et al., 2015).

The SAA3 isoform may also have a role in murine atherogenesis. The overexpression of SAA3 using adeno-associated virus in *Apoe-/-* mice was associated with an increment in atherosclerosis. In addition, the administration of SAA3 antisense oligonucleotides to *Apoe^-/-^ Saa1/2^-/-^* mice reduced aortic root lesion area. Thus, SAA3, a minor acute phase reactant, is pro-atherogenic (Thompson et al., 2018).

### SAA and Monocytosis

Interestingly, the induction of hyperlipidemia in the *Ldlr^-/-^ Saa1/2^-/-^* mice by feeding a Western type diet lead to an increase in total blood monocytes. Most of this increase was attributable to the level of Ly6C^lo^ monocytes and not Ly6C^hi^ monocytes that have been found to be associated with hyperlipidemia induced monocytosis and increased atherosclerosis (Swirski et al., 2007; Krishack et al., 2016). No change in blood monocyte levels or subclass distribution was observed in chow fed *Ldlr^-/-^ Saa1/2^-/-^* mice indicating that the regulation of monopoiesis is the result of an interaction of SAA status and hyperlipidemia. It is notable that the levels of neutrophils and lymphocytes were not altered. An increase in total monocytes, due largely to increased levels of the Ly6C^hi^ monocyte subset, was noted in the bone marrow of the hyperlipidemic *Ldlr^-/-^ Saa1/2^-/-^* animals. This was accompanied by an increase in the monocyte precursor cells macrophage-dendritic progenitor cells (MDP) and its product CDP. Importantly no change was observed in the most primitive precursor cell (HSPC). This is important because this observation, along with the normal neutrophil count, tends to suggest that the increase in monocytes is not the result of a dysfunction in cholesterol homeostasis in the entire hematopoietic system. The reconciliation of the blood monocytosis, especially the higher Ly6C^lo^ monocytes, with the bone marrow findings requires further study. Such questions as (a) whether both subsets of monocytes are released from the bone marrow at similar rates resulting in a higher Ly6C^hi^ monocyte in the blood; (b) whether Ly6C^hi^ monocytes are rapidly converted to Ly6C^lo^ monocytes in the circulation in the presence of low SAA1/2 level; (c) while it is known that Ly6C^hi^ monocytes leave the blood more rapidly than Ly6C^lo^ monocytes, it is not known if and how SAA may influence this process; and (d) does SAA influence the expression and activity of NR4A1, a transcription factor involved in the survival of Ly6C^lo^ monocytes (Hanna et al., 2011). The relative rates of recovery of monocytes and its subsets after clodronate depletion would be of considerable interest in answering these questions.

## The Role of Mimetic Peptides Derived From the Three Apoproteins

### ApoA-I Mimetic Peptides

**FIGURE 2:**
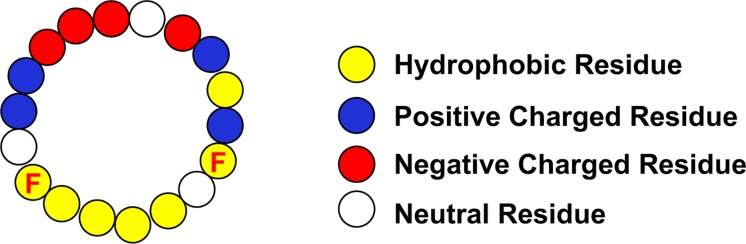
Idealized Apo-I Amphipathic α-Helix. The location of the phenylalanine residues (F) on the hydrophobic face are indicated.

The three apoproteins that are the focus of this review all contain amphipathic α-helical domains. Of these proteins, apoA-I has the most regular repeating helical structure. Mature human apoA-I contains 243 amino acids, with the last 199 amino acids arranged in 10 amphipathic α-helices. Eight of the helices contain 22 amino acids, while the remaining 2 have 11 amino acids. Each helix has a hydrophobic face and a hydrophilic face. Positively charged residues are located at the boundary between the two faces and the negatively charged residues are on the hydrophilic face. Considering the regularity of the amphipathic α-helices in apoA-I, Segrest and colleagues devised an idealized helical peptide from the average components of its eight 22 amino acid helices (Figure 2). This ideal helix contained 18 amino acids, designated 18A, and does not contain the amino acids linking adjacent helices (Anantharamaiah et al., 1985). The helicity and stability of the 18A peptide is increased by N-terminal acylation and C-terminal amidation. The 18A peptide replicated the physical structure of the apoA-I α-helices but does not have any sequence homology to any specific helix. Many variants of the model peptide have been developed which mostly modify the hydrophobic face. As 18A contains 2 phenylalanine residues on its hydrophobic face, it has also been designated 2F. The substitution of the hydrophobic residues valine and leucine in 2F by phenylalanine leads to variants of the peptide designated by the number of phenylalanine residues in the peptide. Up until recently the variant most studied is 4F. As the hydrophilic face contains lysine residues, these peptides synthesized with L-amino acids are susceptible to trypsin-like proteolysis, especially when administered by the oral route. The comparison of various doses of the 4F peptide composed of D-amino acids, and hence resistant to proteolysis, administered either orally or intraperitoneally to mice has generated the hypothesis that a primary site of action of the mimetic peptide is on the small intestine where it modulates the level of bioactive lipids (Navab et al., 2011, 2012). How this relates to macrophage biology in the artery wall is not clear.

Among the atheroprotective functions attributed to apoA-I are its ability to promote cholesterol efflux and participate in multiple steps in reverse cholesterol transport, its anti-oxidative capability due to its ability to sequester oxidized lipids and related to this last function is its capacity to inhibit the chemotaxis of monocytes. Many of the variant mimetic peptides exhibit these functions of apoA-I in *in vitro* assays (Getz et al., 2010). To some extent some of these peptides, by orders of magnitude, are much more powerful on a molar basis than is apoA-I. This is particularly the case for their capacity to bind oxidized fatty acids and phospholipids (Van Lenten et al., 2008b). Both 2F and 4F promote cholesterol efflux (Table 2) and are equally effective in binding macrophage ABCA1 and promoting its stabilization (Tang et al., 2006). The peptides also activate JAK2 autophosphorylation that promotes phosphorylation of STAT3 (Liu and Tang, 2012), resulting in decreased secretion of chemokines and cytokines by macrophages. 4F also reduces lipid rafts and Toll-like receptor 4 levels on the surface of macrophages (Smythies et al., 2010). However, 4F, but not 2F, is atheroprotective (Getz et al., 2010). The intraperitoneal administration of 4F peptide reduces early nascent atherosclerosis in *Apoe^-/-^* and *Ldlr^-/-^* mice (Navab et al., 2002; Wool et al., 2011) but has little effect on mature lesions in 28 week old animals (Wool et al., 2011).

**Table 2:** Apoprotein Mimetic Peptides.

Apoprotein	Mimetic Peptide	Properties
ApoE	ATI-5361 (residues 238–266)	Promotes cholesterol efflux Atheroprotective
	*monomers*	
ApoA-I	2F	Promotes cholesterol efflux
		Binds and stabilizes ABCA1
		No effect on atherosclerosis
	4F	Promotes cholesterol efflux
		Binds and stabilizes ABCA1
		Atheroprotective (early lesions)
	dimers	
	4F-dimers	More active than monomer in promoting cholesterol efflux
	5A (asymmetrical 2F helices)	More active than monomer or symmetrical 2F peptide (37pA) in promoting cholesterol efflux
SAA	SAA2.1 (residues 1–20)	Inhibits acyl cholesterol acyl transferase activity
	SAA2.1 (residues 74–103)	Activates neutral cholesteryl ester hydrolase
		Promotes cholesterol efflux
		Atheroprotective

The studies so far described used monohelical peptides. Since all neighboring helices in intact apoA-I may function co-operatively, tandem amphipathic α-helical peptides have also been studied. Two 18A peptides joined with a single proline residue, designated 37pA, is almost as active as 2F in promoting ABCA1 dependent cholesterol efflux (Tang et al., 2006). But two 4F helices joined by either a single proline or alanine residue or a 7 amino acid sequence derived from the interhelical region between helices 4 and 5 are all more active in facilitating cholesterol efflux than are the monohelical peptides 2F and 4F (Wool et al., 2008). These tandem amphipathic helices are symmetric. However, since the adjacent helices in apoA-I do not have identical sequences, asymmetric tandem peptides have also been studied. Remaley and colleagues generated a peptide, designated 5A, in which the 2F peptide is joined by a single proline residue to a second helix in which 5 of the hydrophobic residues in 2F are replaced by alanine to reduce its lipid affinity (Sethi et al., 2008). This tandem peptide promoted ABCA1 dependent cholesterol efflux with higher specificity than 37pA. Interestingly if the alanine substituted helix is placed at the N-terminal position of the tandem peptide (5A-2 peptide) rather than at the C-terminus its activity in facilitating cholesterol efflux is very much attenuated.

The C-terminal helix (helix 10) of human apoA-I is the helix most responsible for the capacity of the apoprotein to bind lipid (Palgunachari et al., 1996). Ghadiri and colleagues have fashioned a peptide consisting of three copies of this last helix coupled to a bridge scaffold. Even though it is constructed of L-amino acids it is resistant to proteolysis (Zhao et al., 2013, 2014). Indeed, when administered orally to mice, very little peptide is detectable in the plasma, suggesting that like D4F its action may be on the intestine (Wool et al., 2014). This trimeric peptide was incorporated into DMPC nanoparticles for daily administration to *Ldlr^-/-^* mice for 10 weeks along with a Western type diet. This treatment was effective in lowering plasma cholesterol levels, facilitating cholesterol efflux and reducing atherosclerosis in both the whole aorta and the aortic root (Zhao et al., 2014). Interestingly when the monomeric helix was similarly used as nanoparticles for treatment, it was almost as effective as the trimeric peptide.

Other peptides containing the C-terminal helices of human apoA-I are also effective in promoting cholesterol efflux from lipid loaded macrophages. A 33 amino acid peptides containing helix 9, an 11 amino acid helix, with helix 10 (9/10 peptide) or helix 1 (1/9 peptide) are effective in promoting cholesterol efflux (Natarajan et al., 2004). Helix 9 appears to be important for this functionality, though it is not sufficient since the 10/9 peptide with the helices reversed is less effective than the 9/10 peptide and peptides in which helix 9 is joined with other 22 amino acid apoA-I helices are totally ineffective. The apoA-I sequences in inbred strains of mice are not all identical as exemplified in the comparison of apoA-I of the atherosensitive C57BL/6 strain and the atheroresistant FVB/N strain. The proteins differ in their C-termini by two amino acids: Q225K and V226A, C57BL/6 and FVB, respectively. While it is unlikely that these differences play an important role in the relative atherosusceptibility of the two strains, the 9/10 tandem helices of C57BL/6 apoA-I is much more effective in promoting cholesterol efflux from cholesterol loaded macrophages (Sontag et al., 2014).

### ApoE Mimetic Peptides

As discussed above, apoE promotes cholesterol efflux from macrophages. The C-terminus, which contains amphipathic α-helices, is particularly important in its efflux capacity (Vedhachalam et al., 2007). The 26 amino acids encompassing residues 238–266 has been used as the basis for an apoE mimetic peptide designated ATI-5361 (Bielicki et al., 2010; Hafiane et al., 2014). This peptide promotes cholesterol efflux from lipid loaded macrophages *in vitro* and macrophage to feces reverse cholesterol transport *in vivo*. It also reduces atherosclerosis in *Apoe^-/-^* mice. Because this peptide induced muscle toxicity, a variant of this peptide with substitution of phenylalanine for leucine residues and arginine for citrulline residues was generated. CS-6253 and ATI-5367 have similar *in vitro* properties, including the ability to promote cholesterol efflux, but CS-6253 is not toxic *in vivo* (Hafiane et al., 2015).

### SAA Mimetic Peptides

Peptides derived from SAA have also been reported to be able to enhance macrophage cholesterol efflux (Kisilevsky and Tam, 2003; Tam et al., 2005). An N-terminal peptide (residues 1–20) of SAA2.1, but not SAA1.1, inhibits acyl cholesterol acyl transferase in macrophages, while a C-terminal peptide (residues 74–103) of the same isoform activates neutral cholesteryl ester hydrolase. The net result of the modulation of these enzymatic activities is to liberate free cholesterol from the stored cholesteryl esters in the macrophages, which is then available for efflux to acceptors. Similar effects on these two enzymes and cholesterol efflux is observed with acute phase HDL and SAA2.1 liposomes (Tam et al., 2002). The treatment of *Apoe^-/-^* mice with a liposomal formulation of these two peptides reduces and reverses lesion formation (Tam et al., 2005).

## Conclusion

In this review we have discussed the interaction of three HDL apoproteins, apoE, apoA-I and SAA and the mimetic peptides derived from them, with macrophages *in vitro* and *in vivo*. Much of the action of the proteins and peptides is focused on the regulation of macrophage cholesterol homeostasis. But they have other effects, some of which are independent of cholesterol metabolism. Further work is required to distinguish among these various functions and their cellular interactions with respect to the development of atherosclerosis. The peptides offer the opportunity to explore structure-function of apoprotein interactions with the cells and their progenitors, although it has to be realized that not all of the apoprotein properties are replicated by these small molecules. Although much of this review has been concerned with atherosclerosis, it is notable that at least the apoA-I mimetics have been shown to be useful as potential therapies for other inflammatory disorders, such as respiratory inflammations, intestinal inflammation, chronic arthritis, and even some cancer models (Van Lenten et al., 2008a; Cedo et al., 2016).

## Author Contributions

All authors listed have made a substantial, direct and intellectual contribution to the work, and approved it for publication.

## Conflict of Interest Statement

The authors declare that the research was conducted in the absence of any commercial or financial relationships that could be construed as a potential conflict of interest.
